# A Pipeline for Neuron Reconstruction Based on Spatial Sliding Volume Filter Seeding

**DOI:** 10.1155/2014/386974

**Published:** 2014-07-02

**Authors:** Dong Sui, Kuanquan Wang, Jinseok Chae, Yue Zhang, Henggui Zhang

**Affiliations:** ^1^Biocomputing Research Center, School of Computer Science and Technology, Harbin Institute of Technology, Harbin 150001, China; ^2^Department of Computer Science and Engineering, Incheon National University, Incheon 402-751, Republic of Korea; ^3^School of Physics & Astronomy, University of Manchester, Manchester M13 9PL, UK

## Abstract

Neuron's shape and dendritic architecture are important for biosignal transduction in neuron networks. And the anatomy architecture reconstruction of neuron cell is one of the foremost challenges and important issues in neuroscience. Accurate reconstruction results can facilitate the subsequent neuron system simulation. With the development of confocal microscopy technology, researchers can scan neurons at submicron resolution for experiments. These make the reconstruction of complex dendritic trees become more feasible; however, it is still a tedious, time consuming, and labor intensity task. For decades, computer aided methods have been playing an important role in this task, but none of the prevalent algorithms can reconstruct full anatomy structure automatically. All of these make it essential for developing new method for reconstruction. This paper proposes a pipeline with a novel seeding method for reconstructing neuron structures from 3D microscopy images stacks. The pipeline is initialized with a set of seeds detected by sliding volume filter (SVF), and then the open curve snake is applied to the detected seeds for reconstructing the full structure of neuron cells. The experimental results demonstrate that the proposed pipeline exhibits excellent performance in terms of accuracy compared with traditional method, which is clearly a benefit for 3D neuron detection and reconstruction.

## 1. Introduction 

Higher-order cognitive functions in anthropic brain are intricately linked with the processes of nervous system at different biological levels (such as molecular level, cellular level, and system level). The morphological properties of axonal and dendritic arborizations are important aspects of neuronal phenotype. These properties assure the connectivity in the neuron network, thereby facilitate the biological signals transduction in nervous system [1]. Therefore, depicting the function and anatomy structure of neuron cell and networks is of great importance for understanding the way brain works in modern neuron science [2]. Furthermore, great understanding of the mechanism of nervous system can also promote drugs and therapies researching for neurological and psychiatric disease treating.

Extracting neuron morphology from microscopic image data sets is a key point in neurology research. Accurate and efficient reconstruction protocol can facilitate the researches on the function and anatomy structure of neuronal cells and networks. Unfortunately, manually reconstructing neuron structure from microscopy image data sets is labor intensity and time consuming, since the axonal arbors and dendritic are so complex in scale and structure. Therefore, developing new computational methods for neuronal anatomy studying is of particular importance in this context. During the past decades, lots of algorithms and software have been proposed for this task, but most of them achieved limited success.

Since Cohen's team proposed the first fully automated 3D neuron tracing algorithm [3], a large number of approaches have been published for handling the same task in the literature. Generally speaking, these methods can be mainly categorized as minimal path based tracing methods [4, 5], minimum spanning tree methods [6, 7], sequential tracing methods [8, 9], skeletonization methods [3, 10], neuromuscular projection fibers tracing methods [11–16], and active contour based tracing methods [17, 18]. In the minimal path based tracing methods, algorithms were performed in image subregions instead of the entire image, but these methods cannot extract the exact centerline of tubular structures, such as vessels and neuron fibers. In the minimum spanning tree methods, serious of critical seed points were detected firstly and then the detected seeds were linked into tree representation, such as MDL-MST method and k-MST method [6, 7]. The sequential tracing methods were starting from a set of seed points, but the results of these methods were affected by foreground discontinuity, such as gaps and holes, and these defects required additional post- or preprocessing procedures to overcome [8, 9]. The skeletonization methods mainly relayed on a point-spread function based protocol to trace the neuron anatomy structure, but they were also prone to produce loops and spurs which needed extra postprocessing to smooth the noise [10]. Active contour based methods were particularly attractive for neuron tracing and reconstruction was the most employed protocol for this task [17, 18]. Schmitt's group proposed the first active contour tracing method [17], in which the neuron skeleton was parameterized into a 4D snaxels sets that was characterized by its location and radius [17]. But this method needed to manually set some branching, ending, and other critical points. Vasilkoski and Stepanyants [18] proposed a new method for optimizing the tracing based on the active contours. Following that, Roysam's team proposed an open curve snake based tracing method which was broadly applied in this area [19], and it can allow fully automated processing and user control tracing, but this method can only handle distinct edge neuron images data sets and cannot get accurate neuron radius in vague boundary [19]. Beyond that, there still are some automated tracing tools such as Neuromantic [20], Simple Neurite Tracer [21], NeuronJ [22], and a complete list of the tracing tools that can be founded in the survey paper of Meijering [23]. However, most of these tools still need manual assistance to reconstruct the dendritic and axonal arbors. Therefore, automated 3D neurons anatomy tracing tools need a continuous improvement in the future time.

**Figure 1:**
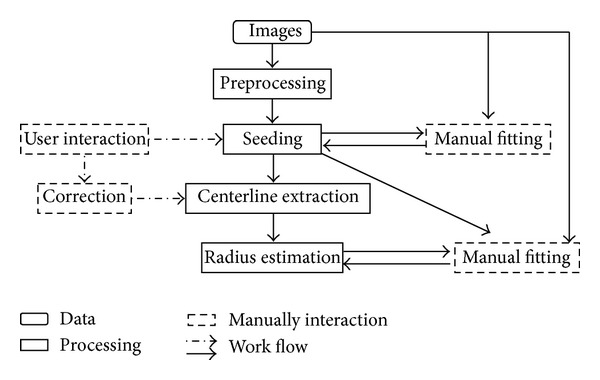
Work flow of neuron tracing pipeline.

Traditionally speaking, the pipeline of tracing was initialized by a serious of preprocessing methods, followed by a critical point detection procedure, which was called seeding. Then, these points were linked by center line extraction method, and, finally, radius estimation was applied to reconstruct the full structure [17]. As depicted in Figure 1, the full pipeline was organized in the work flow. In this pipeline, as a key step, excellent seeding method can assure the accuracy of the following skeletonization. There are two approaches for seeding: (i) segmentation and (ii) filtering. The first one is based on a segmentation process, in which the image volumes covered by the neuron were separated from tissue, such as three-dimensional thinning algorithm, but this method is sensitive to noise. The second approach is using a filter to enhance the line elements. Sato's [24] group proposed a 3D multiscale liner image filter to extract the critical property in medical images. This method employed a combination of eigenvalues in hessian matrix of image intensities. Following this approach, Pizer's group proposed another method based on the concept of cores that detected medial points of the object by correlating opposite boundary points [25]. But most of the seed points detected by these methods were distributed unevenly and located at noncritical position [19].

Radius estimation is another important part in this pipeline, for it is essential for neuron system simulation [23]. Pock's method was greatly accepted in many tracing tasks and was also used in this paper for the tube-like radius estimation.

In this paper, we proposed a new 3D image filter called sliding volume filter (SVF) to enhance the 3D neuron image data sets and then the most listed voxels were chosen as the final seed points. Then, an open curve snake was employed to reconstruct the neuron anatomy structure. Compared with traditional seed detection method, the SVF method could improve the accuracy of neuron anatomy structure in 3D tracing. Finally, radius estimation was applied to the trace the result for the future functional simulation. And the rest of this paper was organized as follows: data sets collection and method design were illustrated in Section 2, the experimental results and discussions were presented in Section 3, and finally the conclusions were drawn in Section 4.

## 2. Methodology

Our works were greatly related to Roysam's pipeline for neuron reconstruction, which was based on open curve snake tracing [19]. In this paper, a SVF was designed for seeding by enhancing the spatial tube-like regions and it could provide seed points for the automatic initialization of open-snake models. At last, Pock's method was applied for radius estimation [23].

SVF was expanded from 2D sliding band filter (SBF). As it was depicted in Quelhas' work [26], the 2D SBF could detect rounded convex region in images. It was firstly introduced for detecting cell center in 2D microscopic images [26]. Recently, our research group employed the SBF to detect cells in section images of cat retinal [27] and another transformed SBF to detect insect cells in light field microscopic images [28]. In 3D volume data sets, a rounded convex region was the same as they were in 2D images in gradient vector distribution and we called it spatial convex region.

### 2.1. Data Sets Used in This Paper

**Figure 2:**
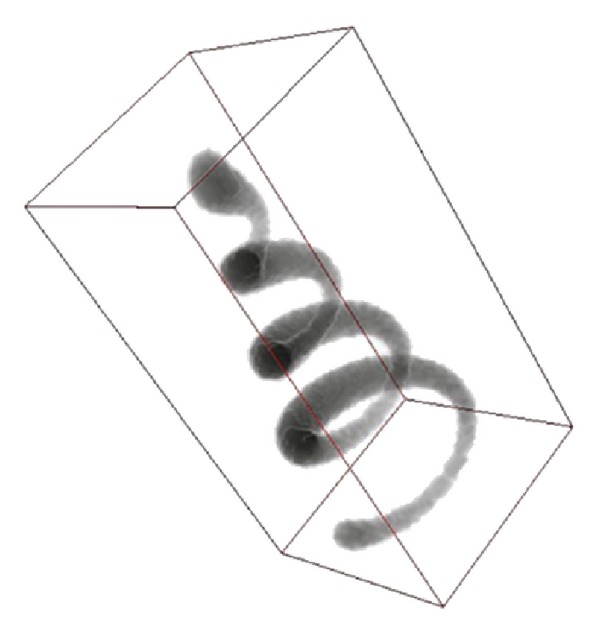
Test data: Helix tube.

**Figure 3:**
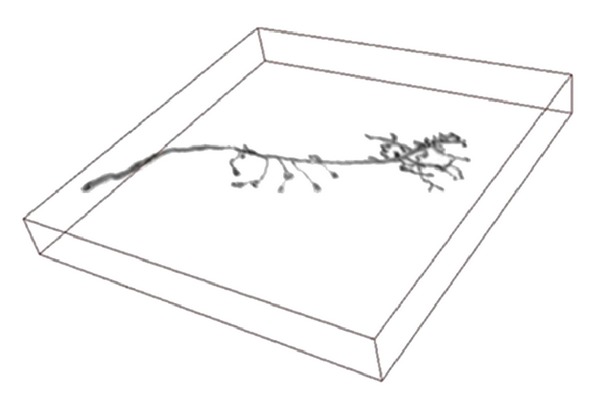
Drosophila olfactory axonal data.

In this paper, we choose two kinds of data sets to validate our proposed seeding method.  Figure 2 shows a helix image volume data which is a classical test data in neuron tracing [19].  Figure 3 is drosophila olfactory axonal image volume data, and this image data set is firstly designed for single cell label and image registration. Both of these data sets were visualized using Ray casting algorithm in our work and all of the tracing algorithms were performed on these volume data sets.

### 2.2. 2D Sliding Band Filter (SBF)

**Figure 4:**
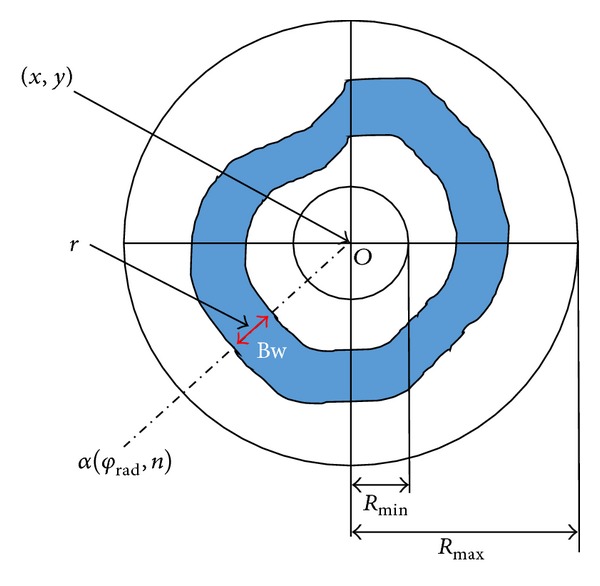
2D sliding band filter (SBF).

To introduce SVF, a concept of 2D SBF is important for understanding. The 2D SBF is a member of Convergence Index (CI) family and firstly introduced for detecting cell center in 2D microscopic images [26]. Unlike most of the liner filters' small support regions (*m* × *m* pixels, where *m* ∈ {2,3, 5…}), the SBF filter has a larger support region. It has a band with fixed width support region, whose position changes in each radius direction and that allows the maximization of the average Convergence Index in the band width.  Figure 4 indicates the support region in SBF and is defined as

(1) SBF(x,y) =1N∑rad=1Nmax⁡Rmin⁡<r<Rmax⁡(1Bw+1∑r−(Bw/2)r+(Bw/2)CI(rad,n)),

where

(2) $$\begin{array}{c} \text{CI}\left(\text{rad},n\right)=\cos\left(\varphi_{\text{rad}}-\alpha \left(\varphi_{\text{rad}},n\right)\right),\\ \varphi_{\text{rad}}=\frac{2\pi \left(\text{rad}-1\right)}{N},\\ \alpha \left(\varphi_{\text{rad}},n\right)=\arctan\left(\frac{\text{Gra}{\text{d}}_{nC}}{\text{Gra}{\text{d}}_{nR}}\right), \end{array}$$

where Grad_*nC*_ and Grad_*nR*_ represent the column and row gradient at image position *n*, *N* represents the number of support region lines irradiate from the center pixel (*x*, *y*), Bw represents the sliding band width, *r* represents the poison of band center in the support region line ranging from *R*_min⁡_ to *R*_max⁡_, and cos⁡(*φ*_rad_ − *α*(*φ*_rad_, *n*)) represents the angle between the gradient vector at (*φ*_rad_, *n*) and the direction of *φ*_rad_.

### 2.3. SVF Seed Detecting

**Figure 5:**
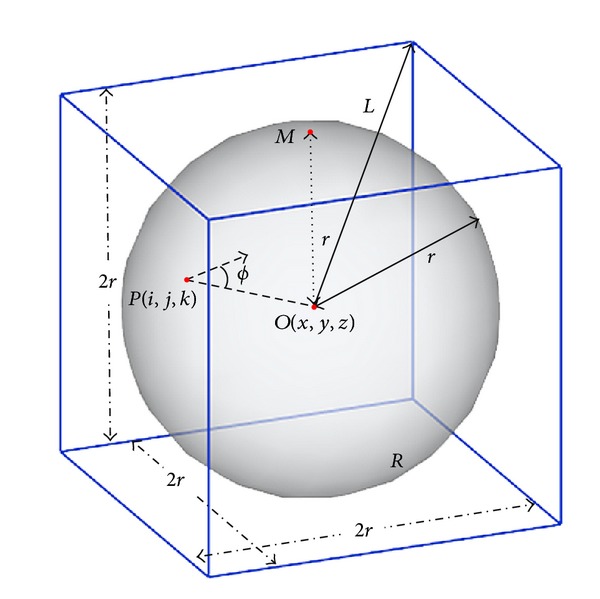
Scheme of spatial convergence index (SCI).

Before the SVF, this part firstly introduces a concept of Spatial Convergence Index (SCI); see Figure 5. Point *O*(*x*, *y*, *z*) is the origin in 3D space and the center of support region *R*. Point *p* is the voxel in support region *R*, and its coordinate relative to *O* is (*i*, *j*, *k*). The radius of the support region *R* is *r*. *ϕ* represents the angle between the gradient vector of *P* and *PO*. And the SCI of *P* relative *O* is defined as follows:

(3) $$\begin{array}{c} \text{SC}{\text{I}}_{PO}\left(i,j,k\right)=\cos\varphi \left(i,j,k\right). \end{array}$$

Then, the SCI of point *O* in the support region is calculated as

(4) $$\begin{array}{c} \text{SC}{\text{I}}_{O}=\frac{1}{N}\underset{p\in R}{\sum }\cos\varphi \left(i_{p},j_{p},k_{p}\right), \end{array}$$

where *N* is the number of voxel in the support region *R*. Based on these concepts, the SVF is defined as

**Figure 6:**
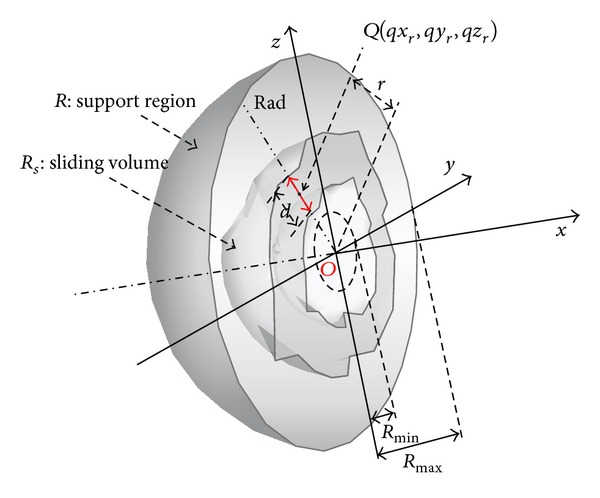
Scheme of sliding volume filter (SVF).

(5) SVF(x,y,z)=1M∗Pn ×∑s=0M−1∑rad=1Pnmax⁡Rmin⁡<r<Rmax⁡(1Vt+1×∑r−(Vt/2)r+(Vt/2)SCIOQs(qxr,qyr,qzr)),

where

(6) $$\begin{array}{c} \text{SCI}\left(x,y,z\right)=\frac{1}{M*P_{n}}\overset{M-1}{\underset{s=0}{\sum }}\underset{(i_{m},j_{m},k_{m})\in R_{s}}{\sum }\cos\varphi \left(i_{m},j_{m},k_{m}\right), \end{array}$$

where *M* represents the section in the support region *R*, *P*_*n*_ represents the support region line in the *s*th section, and *V*_*t*_ represents the thickness of the sliding volume. The scheme of SVF is depicted in Figure 6.

Seed detection is a critical step before the tracing, it can provide seed points for automatic initialization of the open-snake models. In this paper, seed points are detected by SVF filter voxel by voxel from the start position to the end position in the volume data firstly, and then candidate seeds are chosen if they are extreme in the normal plane of the vessel/axon. Detected seeds are then sorted by the SVF response values, from the largest to the smallest value, and created a seed list for tracing.

### 2.4. Neuron Tracing Model

Accurate neuron anatomy structure reconstruction is an import task in neurology. In this part, after initial points selection by the SVF filter, an open-curve snake model for neuron 3D tracing is used for reconstructing the full structure. The open-curve snake is a parametric open curve model. Let *c*(*s*) = (*x*(*s*), *y*(*s*), *z*(*s*)), *s* ∈ [0,1] and let the snake energy to be minimized as

(7) $$\begin{array}{c} E_{\text{total}}=\overset{1}{\underset{0}{\int }}E_{\mathrm{int}}\left(c\left(s\right)\right)+E_{\text{ext}}\left(c\left(s\right)\right)ds, \end{array}$$

where *E*_int⁡_(*c*(*s*)) represents the internal energy for smoothness constraint:

(8) $$\begin{array}{c} E_{\mathrm{int}}\left(c\left(s\right)\right)=\alpha \left(s\right){\left|c_{s}(s)\right|}^{2}+\beta \left(s\right){\left|c_{ss}(s)\right|}^{2}, \end{array}$$

where *α*(*s*) and *β*(*s*) represent the “elasticity” and “stiffness” in the snake, respectively, and

(9) $$\begin{array}{c} E_{\text{External}}=E_{\text{im}}\left(c\left(s\right)\right)+k\left(s\right)gE_{\text{str}}\left(c\left(s\right)\right), \end{array}$$

where

(10) ∇Eim=−∇I¯GVF(x(s),y(s),z(s)),∇Estr(c(s))=−{sign⁡(−cs(s)||cs(s)||·ev1(c(s)))·ev1(c(s))s=0sign⁡(−cs(s)||cs(s)||·ev1(c(s)))·ev1(c(s))s=10s∈(0,1).

In (8), *α*(*s*) and *β*(*s*) are “elasticity coefficient” and “stiffness coefficient,” respectively, in internal energy, and they embedded the regularity of the curve. *α*(*s*) was selected to be 0 for *s* ∈ [0,1], and set *β*(*s*) was selected to be 0 at *s* = 0 and *s* = 1. In (10), the external energy term is employed for making the snake deform along the center line of the neuron fiber, where ∇*E*_im_ is the negative normalized gradient vector flow, *k*(*s*) is a weighted parameter, *ev*_1_(*c*(*s*)) is the first principal direction of the Jacobian matrix, and the ∇*E*_str_(*c*(*s*)) is nonzero item when it is located at the tail and the end pointing to the right direction of the snake.

### 2.5. Radius Estimation

In the following work, to provide more detailed information for functional simulation, the radius estimation is applied to each point on the snake after tracing. As shown in Figure 9, Pock's method is applied for boundaries measurement to detect the tube edge [23]. The edge of the tube-like volume is *B*, and it is defined as a circular centered at *O* which is a seed point, shown in Figure 9. Equation (11) describes the boundary as follows:

(11) B(o,r)=1N∑i=1Ngrad(o+rvai)gmax(−∇I¯GVF(o+rvai)gvai,0),

where *v*_ai_ = cos⁡(ai)*v*_1_ + sin(ai)*v*_2_ is the radial vector in the *v*_1_-*v*_2_ plane of point *O* on the snake and grad(*o* + *rv*_ai_) is the gradient magnitude of the point on the circle. The radius *r* is sampled in the circle by a certain angle distance and in this paper the *N* is set as 8 in the radius circle.

## 3. Results and Discussion

**Figure 7:**
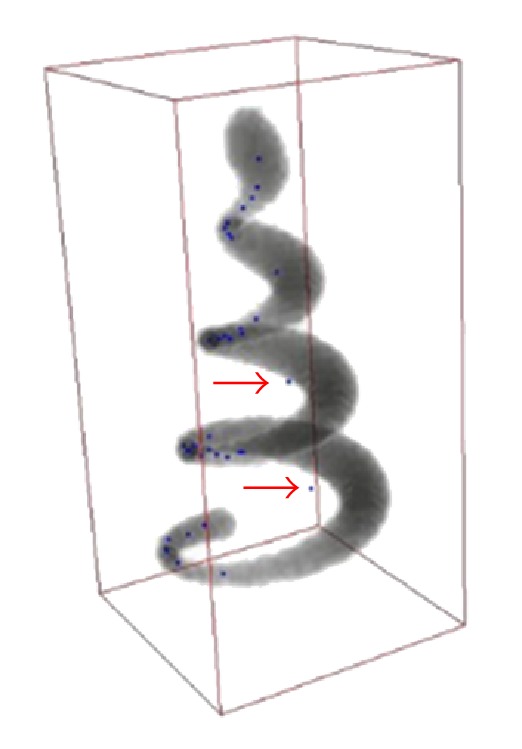
Thresholding seeds detection method.

**Figure 8:**
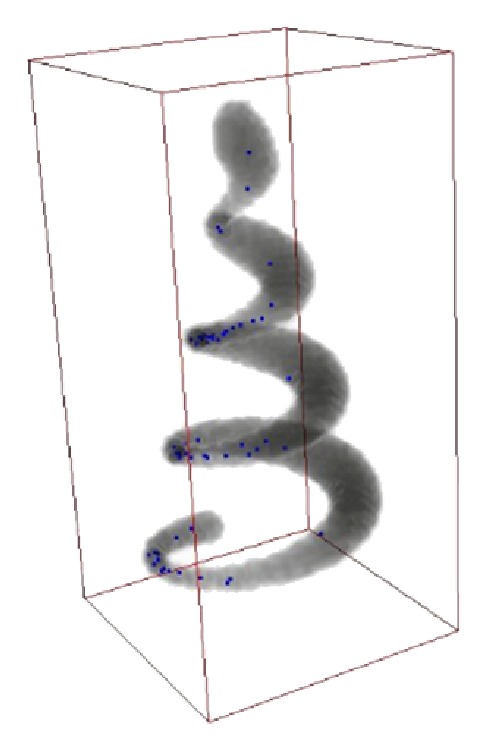
SVF seeds detection method.

**Figure 9:**
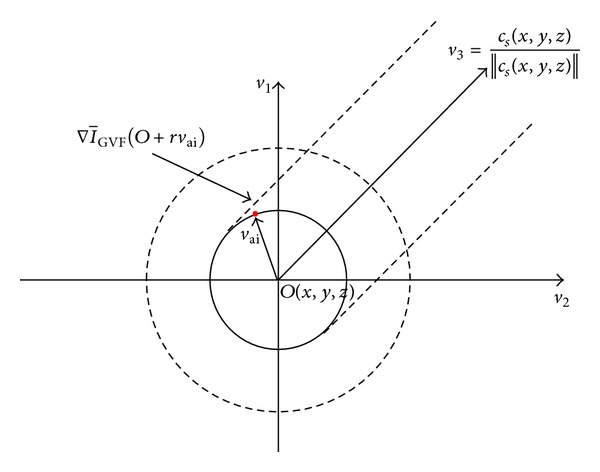
Illustration of the circular cross-section. *v*_3_ is the tangent vector in point *O*, and *v*_1_ and *v*_2_ are the two orthogonal vectors defining the normal plane.

**Figure 10:**
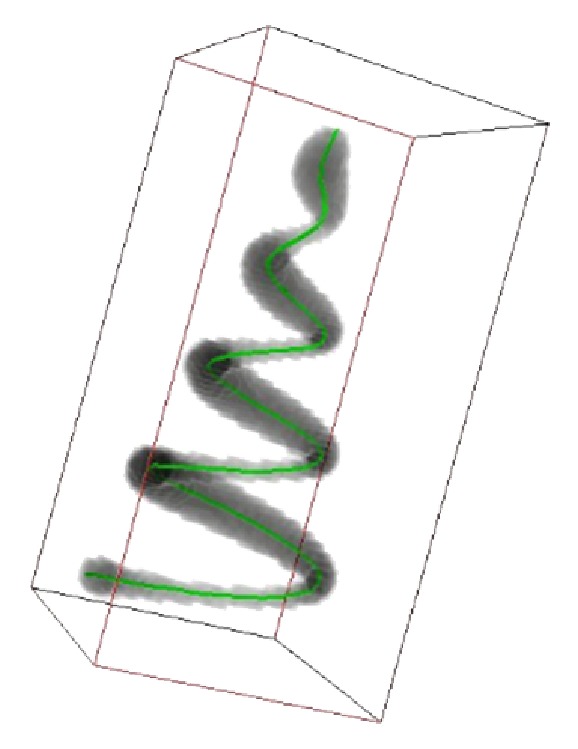
Tracing results by SVF seeds.

For all the image data sets, the following parameters were chosen as the default setting by visual estimation of diameter in average radius of the neuron cross-section. For seeds detection, parameter *M* = 32, *P*_*n*_ = 32 was chosen, respectively, the remaining parameters of SVF were chosen as *V*_*t*_ = 8, *R*_min⁡_ = 10, and *R*_max⁡_ = 30, and the unit of all parameters was “pixels.” Comparison of test data sets seeding results between threshold method and SVF method results has shown an excellent detection results of SVF method in seed detection.  Figure 7 shows a traditional threshold seeding method and there are some seed points that fall out of the edge of real Helix body, which are headlight with red arrows. And Figure 8 is of the same perspective as Figure 7 and shows that the SVF seeding method can detect most of the critical points as candidate seeds. After tracing from the detecting seeds points by SVF, the whole structure of test data sets is generated accurately which are shown in Figure 10.

**Figure 11:**
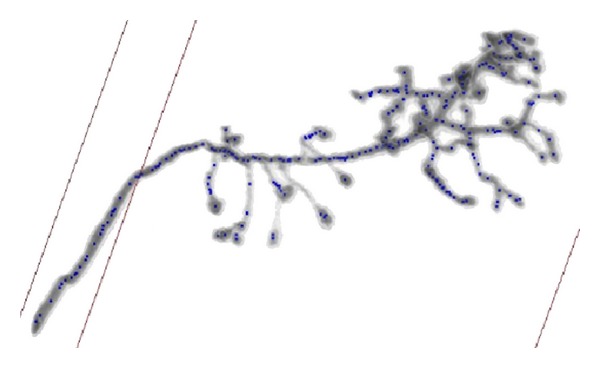
SVF seed detection results for olfactory axonal.

**Figure 12:**
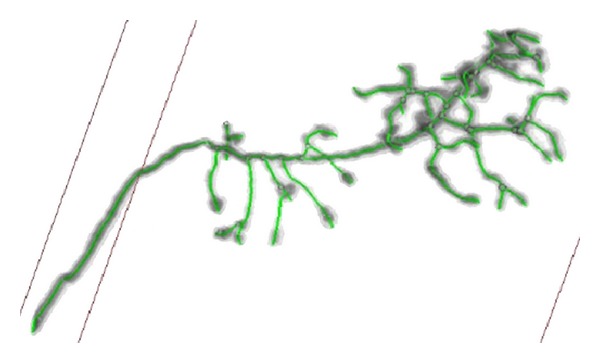
Open curve snake tracing results of olfactory axonal.

When it is applied to real data sets, the SVF seeding method can detect most of the critical seed points in the body of olfactory axonal, shown in Figure 11. Threshold seeding method is not shown here for its poor results. After specifying the branching points, the tracing result of the open curve snake is shown in Figure 12, and it clearly indicates that nearly all of the anatomy structure is reconstructed after tracing.

**Figure 13:**
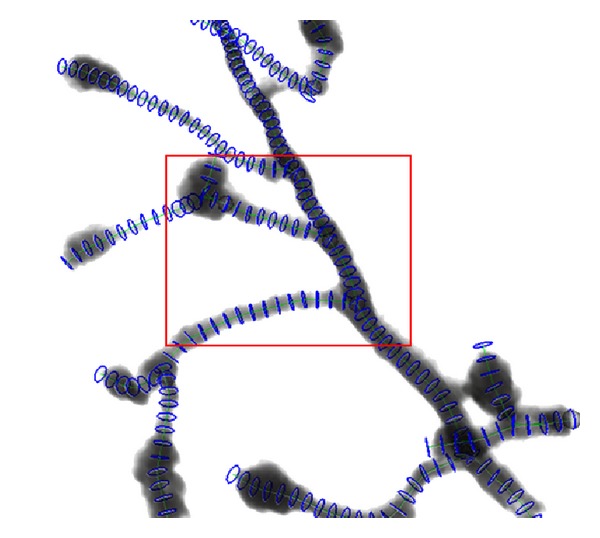
Radius estimation of olfactory axonal.

**Figure 14:**
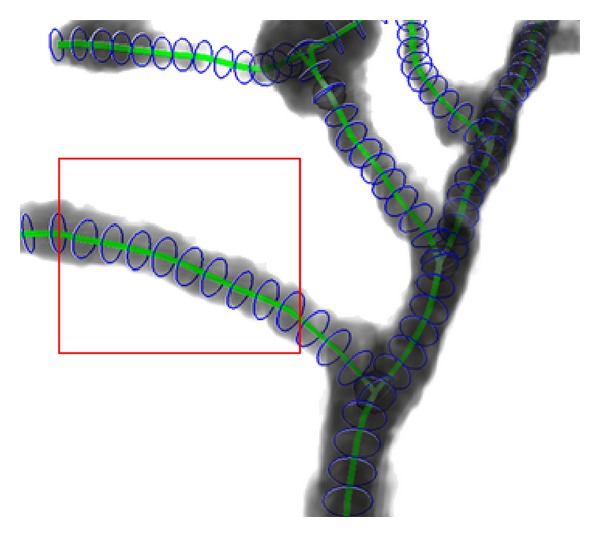
Magnification part of olfactory axonal radius estimation in Figure 11.

**Figure 15:**
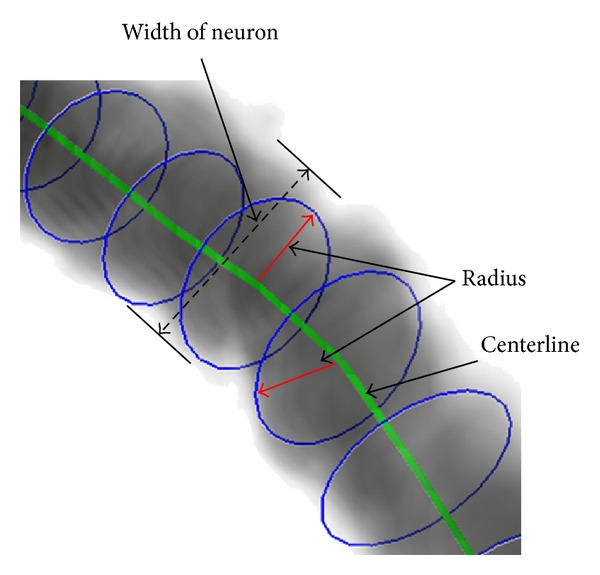
Magnification part of olfactory axonal radius estimation in Figure 12 and detail of radius estimation.

After tracing the full structure, the radius of the olfactory axonal is estimated as a following-up procedure for functional simulation. As shown in Figure 13, the gray and black area represents the body of the olfactory axonal, the green line represents the central line of the olfactory axonal, and the blue ring represents the radius of each part from the central line, and it depicts that most of the radius is estimated by the method.  Figure 14 exhibits a magnification of the red rectangle area in Figure 13. And another magnification of red rectangle area in Figure 14 is shown in Figure 15.

In Figure 15, the width of olfactory axonal, estimated radius, and center line are marked separately. This task is for the future functional neuronal simulation which is not discussed in this paper.

## 4. Conclusion

In this paper, a novel seeding method based on spatial SVF is proposed for neuron reconstruction from microscopic image data sets which were collected by confocal microscopy. The seeding results comparison shows that the SVF method can detect seed points accurately in test data sets and detect most of the critical points in olfactory axonal data sets. After open curve snake tracing, both of the data set's structures are reconstructed from SVF seeds. In the last part of our work, a radius estimation method is applied to the tracing result for future functional simulation.

Finally, it is worth noting that this method can clearly be a benefit for seeding task in the protocol of neuron tracing. However, uneven illumination produced by a microscope is also a critical factor influenced the seeding accuracy. Therefore, some illumination correction methods will be studied to improve our method in the future works.
